# The anti-tumor effect of shikonin on osteosarcoma by inducing RIP1 and RIP3 dependent necroptosis

**DOI:** 10.1186/1471-2407-13-580

**Published:** 2013-12-06

**Authors:** Zeze Fu, Biyong Deng, Yuxin Liao, Liancheng Shan, Fei Yin, Zhuoying Wang, Hui Zeng, Dongqing Zuo, Yingqi Hua, Zhengdong Cai

**Affiliations:** 1 Department of Orthopedics, Shanghai Tenth People’s Hospital, Tongji University, School of Medicine, Shanghai 200072, China; 2 Postdoctoral Research Station of School of Life Science and Technology of Tongji University, Shanghai 200092, China

**Keywords:** Osteosarcoma, Necroptosis, Shikonin, Metastasis, RIP1, RIP3

## Abstract

**Background:**

Osteosarcoma is the most frequent primary malignant bone tumor, notorious for its lung metastasis. Shikonin, an effective constituent extracted from Chinese medicinal herb, was demonstrated to induce necroptosis in some cancers.

**Methods:**

MTT assay was performed to detect cell survival rate in vitro. Flow cytometry was used to analyze cell cycle and cell death. Western blot was performed to determine the expression levels of RIP1, RIP3, caspase-3, caspase-6 and PARP. The tibial primary and lung metastatic osteosarcoma models were used to evaluate the anti-tumor effect of shikonin in vivo.

**Results:**

The cell survival rate was decreased in a dose and time dependent manner when treated with shikonin. No major change in cell cycle was observed after shikonin treatment. The cell death induced by shikonin could be mostly rescued by specific necroptosis inhibitor necrostatin-1, but not by general caspase inhibitor Z-VAD-FMK. The number of necrotic cells caused by shikonin was decreased after being pretreated with Nec-1 detected by flow cytometry in K7 cells. After 8-hour treatment of shikonin, the expression levels of RIP1 and RIP3 were increased while caspase-3, caspase-6 and PARP were not activated in K7 and U2OS cells determined by Western blot. Size of primary tumor and lung metastasis in shikonin treated group were significantly reduced. The protein levels of RIP1 and RIP3 in primary tumor tissues were increased by shikonin. The overall survival of lung metastatic models was longer compared with control group (p < 0.001).

**Conclusions:**

Shikonin had prompt but profound anti-tumor effect on both primary and metastatic osteosarcoma, probably by inducing RIP1 and RIP3 dependent necroptosis. Shikonin would be a potential anti-tumor agent on the treatment of primary and metastatic osteosarcoma.

## Background

Osteosarcoma is the most common primary malignant bone tumor accounting for approximately 60% of all bone sarcoma [1,2]. With the advance of chemotherapy, although the long-term cure rate after surgery for non-metastatic osteosarcoma has risen from 25% to 60% [3], the survival rate for osteosarcoma is still rather low. Most osteosarcomas are high grade with part of them were accompanied by lung metastasis [4]. Metastatic disease is usually not sensitive to conventional chemotherapy with long-term survival rate approximately 20% [5]. Therefore, the development of chemotherapy for osteosarcoma is urgently needed.

For a long time, apoptosis was regarded as the sole form of programmed cell death, while necrosis was considered as an unregulated and uncontrollable process. In 2004, Zong, WX, et al. found a regulated form of necrotic cell death during the damage of DNA [6], which was named as “necroptosis” later and suggested that necrosis might not be absolutely unregulated. In 2005, Degterev, A, et al. found that Nec-1 (necrostatin-1) was a specific inhibitor of necroptosis [7]. The idea of “necroptosis” was demonstrated by a series of subsequent studies in which increasing signal molecules functioning as initiators or effectors of necroptosis such as receptor-interacting protein 1 [8] (RIP1, RIPK1) and receptor-interacting protein 3 [9,10] (RIP3, RIPK3) or inhibitors such as necrostatin-1 (Nec-1), were discovered. Since necroptosis is a pathway separate from apoptosis, all the barriers set up in cancer cells to avoid apoptosis are no longer problems for necroptosis [11].

Shikonin, an effective constituent, purified from *Lithospermum erythrorhixon*, a Chinese medicinal herb, was widely used in anti-inflammatory process [12,13]. Shikonin was thought to have anti-tumor effect by inducing apoptosis until people found that shikonin could circumvent cancer drug resistance by inducing necroptosis in 2007 [11,14]. Interestingly shikonin also exert two death modes of apoptosis and necroptosis in KL-60 cells depending on its concentrations [15]. Moreover, shikonin was demonstrated to mediated necrotic cell death via a RIP1-RIP3 complex similar to TNFα-directed necrotic cell death, and this pronecrotic complex was blocked by a reactive oxygen species (ROS) scavenger or Nec-1 concomitantly with protection against cell death [16]. In 2011, the first molecular target of shikonin was reported in which shikonin played a role in the anti-tumor effect by inhibiting pyruvate kinase-M2(PKM2). PKM2 is universally over expressed in cancer cells and dictated to the last rate-limiting step of glycolysis vital for cancer cell proliferation [17]. Recently, shikonin was also found to be a cytotoxic DNA-binding agent [18]. Furthermore, shikonin and its analogs were demonstrated hardly to inducer cancer drug resistance [19]. The effect of shikonin on bone sarcomas is still unclear. In this study, we tested whether shikonin had anti-tumor effect on osteosarcoma and explored the underlying mechanism.

## Methods

### Cell Lines and culture

Murine osteosarcoma cell lines K7, K12 and K7M3 cell lines were from Dr. Kleinerman’s lab in MD Anderson Cancer Center which were originally established by Khanna [20]. Human osteosarcoma cell lines U2OS and 143B cell lines were obtained from American Type Culture Collection (ATCC). All cells were cultured in high glucose Dulbecco’s Modified Eagle’s Medium (DMEM-h; Thermo, America) supplemented with 10% fetal bovine serum (Thermo, America), 100 U/ml penicillin and 100 μg/ml streptomycin (Thermo, America) in a humidified incubator at 37°C in 5% CO_2_.

### Drugs and antibodies

Purified shikonin (>98%) was purchased from Shanghai Tauto Biotech Co., Ltd. Stock solution at 50 mM was made in dimethyl sulfoxide (DMSO; Sigma, America) and stored in the dark at −20°C. The final shikonin concentrations used for different experiments were prepared by diluting the stock solution with DMEM-h. The antibodies used for Western blot were as follows: rabbit anti-Actin (Santa Cruz, CA, USA), anti-caspase-3 (Cell Signaling Technology Inc., Danvers, MA), anti-caspase-6 (Cell Signaling Technology Inc., Danvers, MA), anti-PARP (Cell Signaling Technology Inc., Danvers, MA) and mouse anti-RIP1 (BD, CA, USA), anti-RIP3 (Abcam, USA).

### MTT assay

Cells were seeded into 96-well plates (cultured overnight for adherent cells) and treated with shikonin at a series of concentrations (0, 1, 3, 5, 7.5, 10, 12.5, 15 μΜ) for 8 hours or treated with shikonin (3 μΜ) for 8, 16 or 24 hours. Cells incubated with DMEM-h were regarded as control group. After 8, 16 or 24-hour incubation, 20 μl MTT (5 mg/ml, Sigma, America) was added into each well for another 4-hour incubation. After that, the supernatant was removed and 150 μl DMSO was added into each well in order to solubilize the blue-purple crystals of formazan. The absorbance was then measured using a model ELX800 Micro Plate Reader (Bio-Tec Instruments, Inc.) at 490 nm. The survival rate was calculated according to the following formula: Survival rate = Absorbance of treatment / Absorbance of control × 100%.

In the inhibition experiment, K7, K12, K7M3 and U2OS cells were treated with shikonin (3 μΜ) while 143B cells were treated with shikonin (6 μΜ) in the absence or presence of necrostatin-1 (Nec-1; Sigma, America) or Z-VAD-FMK (Sigma, America) for 8 hours. The cell survival rate was measured by MTT assay. When added MTT, the supernate in the well with Nec-1 was discarded and added DMEM-h again.

### Flow cytometry analysis

Osteosarcoma cells (K7, K12, K7M3, U2OS and 143B) were plated in 6-well plates and synchronized with DMEM-h containing 10% fetal bovine serum. After 8-hour incubation, control cells (incubated with DMEM-h) and shikonin-treated cells (143B cells were treated with 6 μΜ shikonin while other cells were treated with 3 μΜ shikonin) in the presence or absence of Nec-1 (50 μΜ) were collected, washed twice in cold PBS. The cells used in cell cycle were mixed in 300 μl of 1× binding buffer, and incubated at room temperature for 15 min with propidium dide (PI, Sigma, America), NP-40, and RnaseA (BD Biosciences) while the cells used in cell death were mixed in 100 μl of 1× binding buffer, and incubated at room temperature for 15 min with an annexin-V/PI (BD Biosciences) double staining solution. Stained cells were analyzed by flow cytometry. The percentage of cells in the different stages and the percentage of necrosis cells were calculated using ModFit LT software (Verity Software House).

### Western blot assay

K7, U2OS and 143B cells were treated with different concentrations of shikonin for 8 hours. Cells were washed twice with PBS solution, lysed with RIPA Lysis Buffer (Beyotime Institune of Biotechnology, Shanghai, China) and protease inhibitor (Thermo scientific). Tumor tissues were retrieved from −80°C storage and immersed rapidly in liquid nitrogen. The resulting powder was lysed with RIPA Lysis Buffer and protease inhibitor. Protein concentrations were determined with Pierce BCA Protein Assay Kit (Thermo Scientific). Equivalent amounts of total protein (50 μg) were boiled and electrophoretically seperated on a 10% polyacrylamide gel at 80 volts. The proteins were transferred to a nitrocellulose filter membrane. Membranes were blocked for 60 min with 5% milk solutions prepared in PBS, incubated overnight at 4°C with 1:1000 dilutions of the primary antibodies (RIP1, RIP3, PARP, caspase-3, caspase-6 and Actin), washed three times for 10 min each time with Tween 20 (1:1000 dilution)-PBS, incubated for 1 hour with the appropriate peroxidase-conjugated secondary antibody (1:1000 dilution). Membranes were washed with Tween 20-PBS three times for 10 min each and were developed using the Odyssey two-color infraed laser imaging system. The signal generated by Action was used as an internal control.

### Animal experiments

Animal experiments were performed on 4-week-old female mice (BALB/C). Mice were housed in a standard animal laboratory with free access to water and food. They were kept under constant environmental conditions with a 12-hour light–dark cycle. All operations were performed under aseptic conditions. All the animal-related procedures were approved by the Animal Care and Use Committee of The Tenth People's Hospital of Shanghai. This study was also approved by the Science and Technology Commission of Shanghai Municipality (ID: SYXK 2007–0006) with the permit number 2011-RES1.

### Mice tibial tumor models and treatment regimen

Balb/c mice (4 weeks of age) were purchased from Shanghai Slac Laboratory Animal Co., Ltd. K7 cells were digested and washed by cold PBS for three times, suspended in cold PBS. The final concentration of K7 cells was 1 × 10^8^/ml. The cell suspension (10 μl) was injected into medullary cavity of tibia. Mice were divided into two groups, shikonin group and control group (each had 10 mice). Three weeks later, when the tumors in the tibia were macroscopic, shikonin group was injected with shikonin (2.0 mg/kg, diluted with 5% DMSO) while control group was injected with 5% DMSO. Both groups were injected intraperitoneally every other day for seven times in all. The mice were euthanized two days after the last injection. The primary tumor size and lung metastasis were observed. Posterior limb with tumors and lungs were weighted. Necrotic degree of primary tumors and lung metastasis was detected by HE stain. The expression levels of RIP1 and RIP3 in primary tumor tissues were determined by Western blot.

### Mice lung metastatic models and treatment regimen

Balb/c mice (4 weeks of age) were purchased from Shanghai Slac Laboratory Animal Co., Ltd. K7 cells were digested and washed by cold PBS for three times, suspended in cold PBS. The final concentration of K7 cells was 5 × 10^6^/ml. The cell suspension (100 μl) was injected into the mice from caudal vein. Two weeks later, mice were divided into two groups (each had 10 mice), experimental group was injected with shikonin (2.0 mg/kg, diluted with 5% DMSO), control group was injected with 5% DMSO. Both groups were injected intraperitoneally every other day.

### Statistical analysis

Statistical analysis was performed using GraphPad Prism 5 (La Jolla, CA, USA). All measurement data were expressed as mean ± standard deviation (SD), and compared between two groups using Student’s t test. P < 0.05 was considered statistically significant.

## Results

### Shikonin had prompt killing effect on osteosarcoma cells

**Figure 1:**
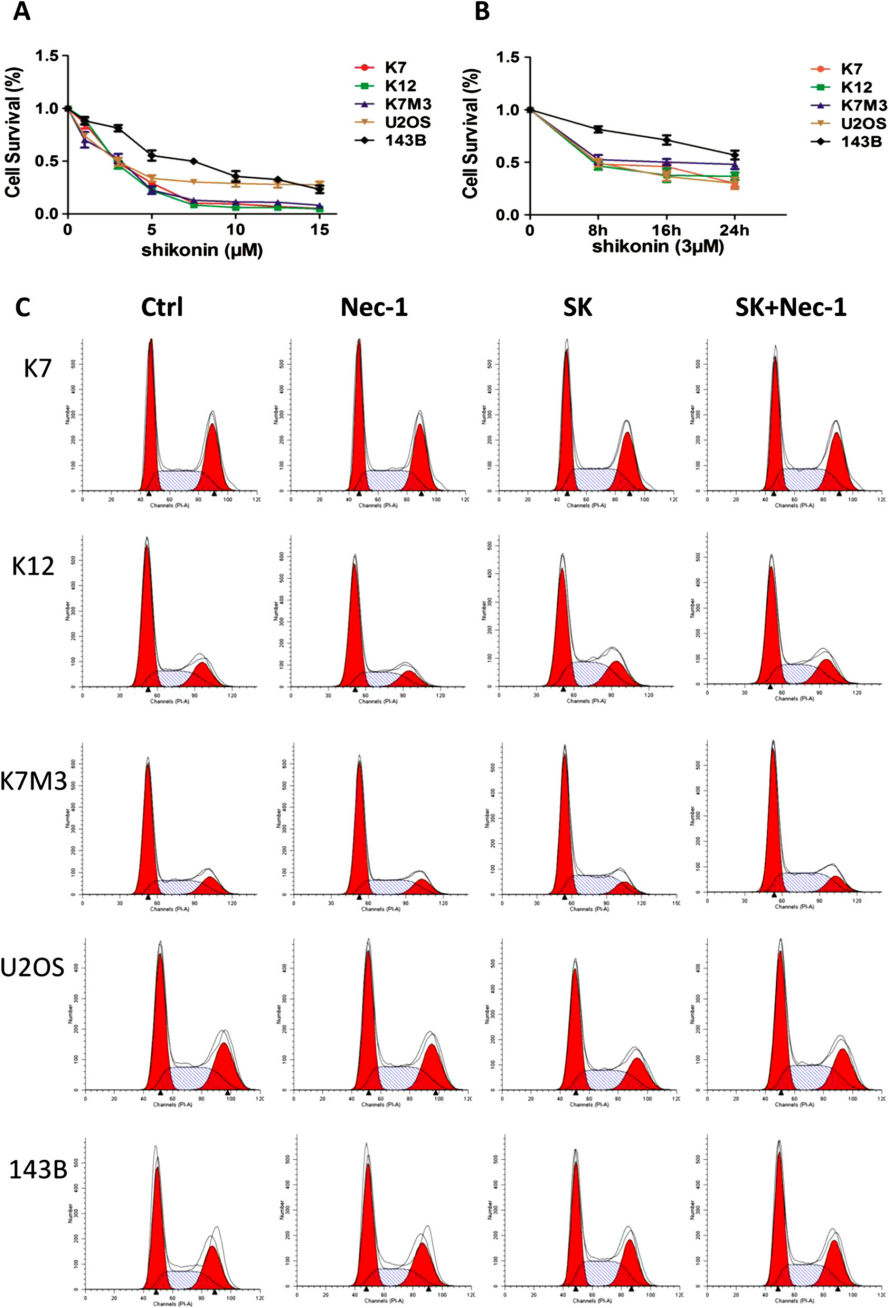
**The prompt anti-tumor effect of shikonin on osteosarcoma cells. (A)** Osteosarcoma cells (K7, K12, K7M3, U2OS, 143B) were treated with shikonin in different concentrations (0, 1, 3, 5, 7.5, 10, 12.5, 15 μΜ) for 8 hours. The cell survival rate was measured by MTT assay. **(B)** Osteosarcoma cells (K7, K12, K7M3, U2OS, 143B) were treated with shikonin (3 μΜ) for 8, 16 or 24 hours. The cell survival rate was measured by MTT assay. **(C)** K7, K12, K7M3, U2OS cells were treated with shikonin (3 μΜ) while 143B cells were treated with shikonin (6 μΜ) in the absence or presence of Nec-1 (50 μΜ) for 8 hours. Nec-1 was pretreated for 2 hours before shikonin. All osteosarcoma cells had no significant changes in G0/G1, G2/M and S phases. Data are representative of 3 independent experiments.

We firstly evaluated the cytotoxity of shikonin on osteosarcoma cells in vitro. Cells were treated with shikonin in different concentrations for 8 hours. The IC_50_ of K7, K12, K7M3, U2OS, 143B was 2.87, 2.72, 3.02, 3.18, 6.45 μΜ respectively at 8-hour treatment of shikonin (Figure 1A). The cell survival rate decreased time-dependently with the treatment of shikonin (3 μΜ) for 8, 16 and 24 hours (Figure 1B). The decrease of cell viability happened in a quick fashion within the first 8-hour in osteosarcoma cells. Conventional chemotherapy agent such as cisplatin and doxorubicin showed almost no cell killing effect at 8-hour theatment in IC_50_ dosage (data not shown).

We then tested the cell cycle change after shikonin treatment of osteosarcoma cells (K7, K12, K7M3, U2OS, 143B). There was no significant change in cell cycle after being treated with shikonin for 8 hours in the absence or presence of Nec-1 detected by flow cytometry (Figure 1C). All these data suggested that shikonin had very prompt but profound cell killing effect on osteosarcoma cells.

### Shikonin induced necroptosis in osteosarcoma cells

**Figure 2:**
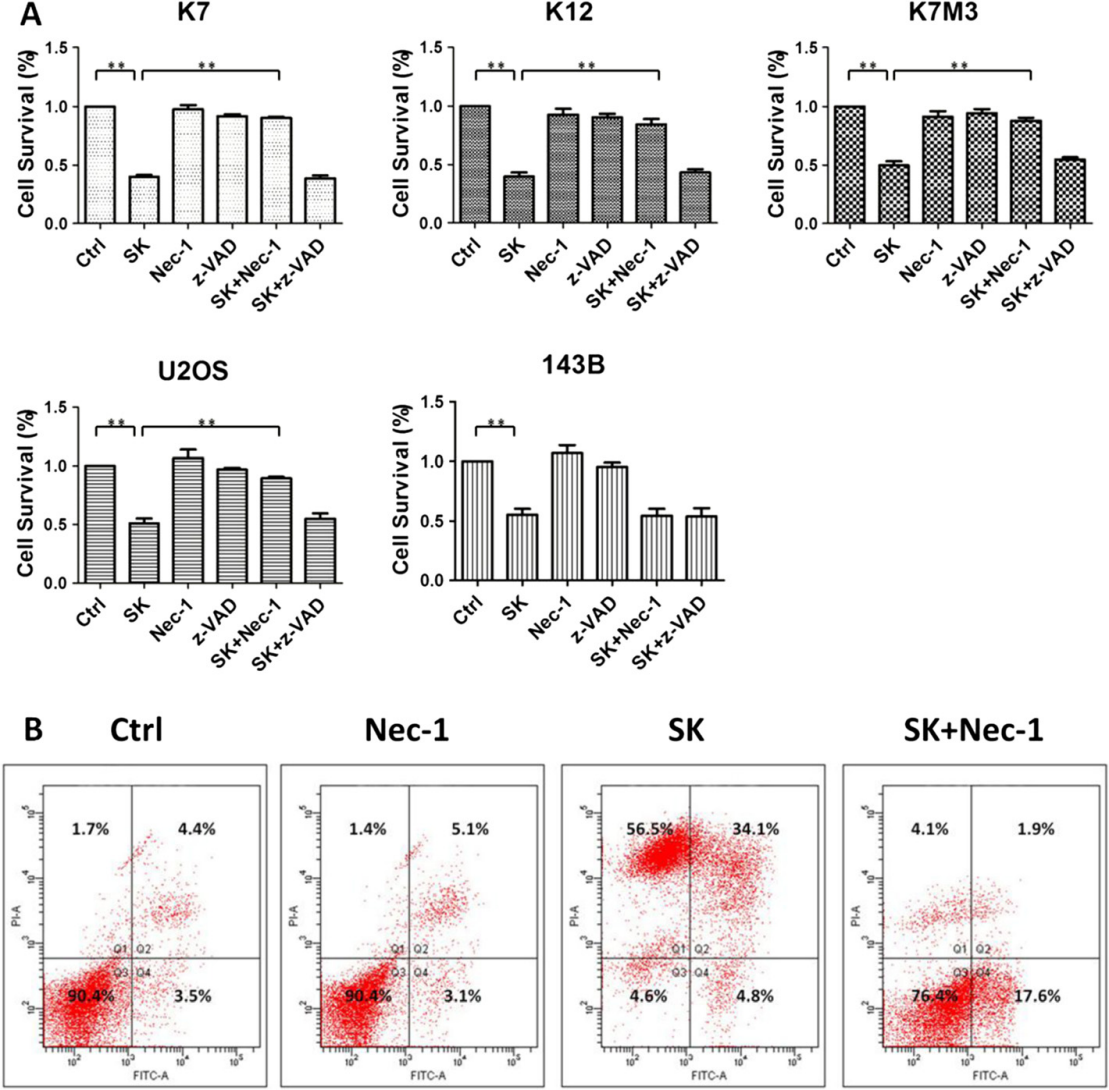
**The induction of necroptosis in osteosarcoma cells by shikonin. (A)** K7, K12, K7M3, U2OS cells were treated with shikonin (3 μΜ) while 143B cells were treated with shikonin (6 μΜ) in the absence or presence of Nec-1 (50 μΜ) or Z-VAD-FMK (20 μΜ) for 8 hours. Nec-1 and Z-VAD-FMK were pretreated for 2 hours before shikonin. The cell survival rate was determined by MTT assay as described in Methods. ** represents p < 0.01. **(B)** K7 cells were treated with shikonin (3 μΜ) in the absence or presence of Nec-1 (50 μΜ) for 8 hours. Nec-1 was pretreated for 2 hours before shikonin. After treatment of shikonin, PI + cells increased significantly. When added with Nec-1 together with shikonin, the PI + cells induced by shikonin decreased significantly. Data are representative of 3 independent experiments.

To explore the mechanism of how shikonin kill osteosarcoma, we added apoptosis inhibitor and necroptosis inhibitor prior to shikonin treatment. After 8-hour incubation of shikonin, the survival rate of K7, K12, K7M3, U2OS and 143B cells was reduced to 40.03 ± 2.6, 39.86 ± 3.6, 49.73 ± 3.5, 51.08 ± 4.1, 55.21 ± 5% respectively, all differently from that of control group (p < 0.01). After pretreated with Nec-1 before adding shikonin, the corresponding survival rate was increased to 90.25 ± 1.7, 84.58 ± 4.6, 87.98 ± 2.5, 89.38 ± 1.5% in K7, K12, K7M3 and U2OS cells respectively (p < 0.01). However, the similar increase of survival rate was not obvious for 143B cells (Figure 2A). Cell death caused by shikonin could not be rescued by Z-VAD-FMK in 143B cells. The death caused by shikonin in K7 cells was detected by flow cytometry showed in Figure 2B. K7 cells was incubated with shikonin (3 μΜ) for 8 hours in the absence or presence of Nec-1 (50 μΜ), which was pretreated for 2 hours before shikonin. The percentage of PI positive cells increased from 6.53 ± 0.45% to 88.5 ± 2.05% (p < 0.001) when treated with shikonin (3 μΜ) which were decreased to 7.03 ± 1.00% (p < 0.001) in the presence of Nec-1 (50 μΜ) (Figure 2B). All these findings evidently showed that shikonin is a potent necroptosis inducer in osteosarcoma.

### Shikonin induced necroptosis via upregulating RIP1 and RIP3

**Figure 3:**
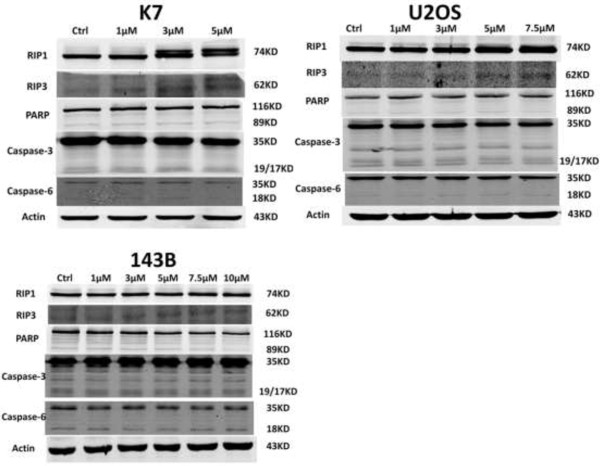
**The induction of necroptosis by shikonin via upregulating RIP1 and RIP3 in K7 and U2OS cells.** K7, U2OS and 143B cells were treated with different concentrations of shikonin for 8 hours. The expression of RIP1, RIP3, caspase-3, caspase-6 and PARP was detected by Western blot. The expression of the necroptosis related proteins, RIP1 and RIP3, was increased while apoptosis related proteins, caspase-3, caspase-6 and PARP were not activated in K7 and U2OS cells after being treated with shikonin. The expression of RIP1 and RIP3 had no obvious changes and caspase-3, caspase-6 and PARP were not activated in 143B cells after being treated with shikonin. Data are representative of 3 independent experiments.

RIP1 and RIP3 were regarded as crucial modulator of necroptosis. As showed in Figure 3, the protein levels of RIP1 and RIP3 were significantly increased in K7 and U2OS cells after shikonin treatment for 8 hours in a concentration dependent manner. However, caspase-3, caspase-6 and PARP, indicators for apoptosis, were hardly activated after being treated with shikonin for 8 hours in neither K7 nor U2OS cells. Interestingly, the expression of RIP1 and RIP3 had no obvious change and caspase-3, caspase-6 and PARP were not activated in 143B cells after shikonin treatment. These data indicated that the main mechanism for shikonin in causing cell death in osteosarcoma is to induce RIP1 and RIP3 dependent necroptosis, independent of apoptosis.

### Shikonin had anti-tumor effect on primary and metastatic osteosarcoma by inducing necroptosis

**Figure 4:**
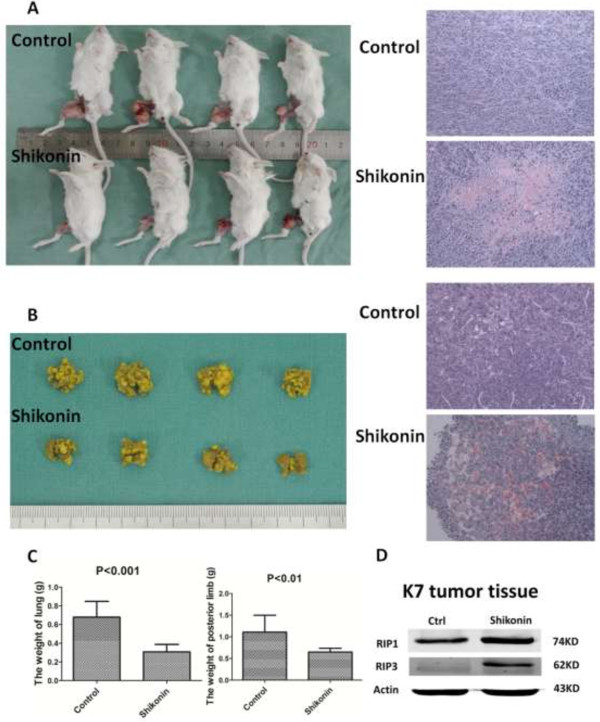
**The anti-tumor effect of shikonin on primary and metastatic osteosarcoma by inducing necroptosis in vivo. (A)** Macroscopic appearance of tibial primary osteosarcoma tumors in Balb/c mice after treatment with shikonin or 5% DMSO. The tumor size in shikonin group was smaller than that in control group. The HE stain of primary tumors showed that the degree of tumor necrosis in shikonin group was higher than that in control group. **(B)** Macroscopic appearance of lung got from tibial primary models. The lung metastaseis in shikonin group were less than that in control group. The HE stain of lung matastasis also showed that the degree of lung matastatic tumur necrosis in shikonin group was higher than that in control group. **(C)** The posterior limbs with primary tumor in shikonin group were lighter than that in control group (p < 0.01) while the lung in shikonin group was lighter than that in control group (p < 0.001). **(D)** The expressional level of RIP1 and RIP3 increased in shikonin group in primary tumor tissues compared with that in control group detected by Western blot. Data in D are representative of 3 independent experiments.

To evaluate the anti-tumor effect of shikonin in vivo, an orthotopic osteosarcoma model was established by intratibial injection of K7 cells. The mice (n = 10) were injected with shikonin (2.0 mg/kg) while control group (n = 10) were injected with 5% DMSO intraperitoneally every other day for seven times in all. The general condition of mice, e.g. alertness and physical activity, was observed to be normal during the whole experiment in both groups. The mice were euthanized two days after the last treatment. The results showed that the tumor size in shikonin treated group was smaller compared with control group (Figure 4A) and the weight of posterior limb with tumors in shikonin treated group (0.65 ± 0.09 g) was lighter compared with control group (1.11 ± 0.39 g) significantly (p < 0.01), which both reflected the inhibition of tumor growth with shikonin. The HE stain of primary tumors showed that the degree of tumor necrosis in shikonin group was higher compared with control group (Figure 4A). The protein levels of RIP1 and RIP3 in primary tumor tissues gained from the mice were significantly increased compared by shikonin treatment (Figure 4C).

Since osteosarcoma mainly metastasizes to the lung, mouse lungs were also harvested for examination. The number of lung metastasis was significantly reduced with shikonin treatment compared with control group (Figure 4B) and the weight of lung in shikonin group (0.31 ± 0.08 g) was lighter compared with control group (0.68 ± 0.17 g) significantly (p < 0.001). The HE stain of lung metastasis also showed that the degree of tumor necrosis in shikonin group was higher compared with control group (Figure 4B).

### Shikonin prolonged the survival of metastatic disease

**Figure 5:**
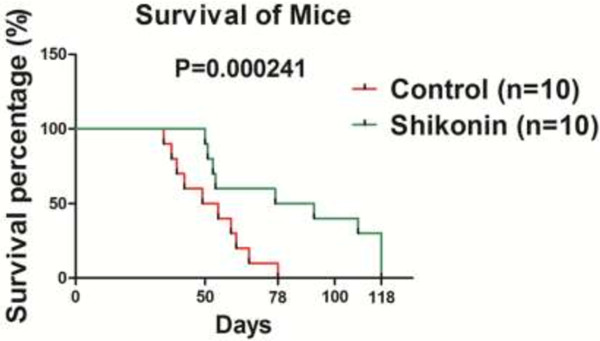
**Survival of mice treated with DMSO and shikonin respectively.** The lung metastatic models were constructed by osteosarcoma K7 cells in 4-week-old female Balb/c mice. The experimental group (n = 10) was injected with shikonin (2.0 mg/kg, diluted with 5% DMSO) while control group (n = 10) was injeced with 5% DMSO intraperitoneally every other day. The survival time in shikonin group was longer than that in control group significantly (p < 0.001).

In order to test the effect of shikonin on metastatic osteosarcoma, the mice lung metastasis models were established by i.v injection of K7 cells. The mice in experimental group (n = 10) were injected with shikonin (2.0 mg/kg) while control group (n = 10) were injected with 5% DMSO intraperitoneally every other day. Experiment was ended 118 days later when 3 mice in experiment group were alive, verse all mice died in control group. The survival time in experimental group was significantly prolonged compared with control group (p < 0.001) (Figure 5). These data suggested that shikonin is effective against metastatic lesions in the lung, and possibly could be developed into salvage treatment for late stage osteosarcoma patients.

## Discussion

In this study, we used different cell lines to test the efficacy of shikonin on osteosarcoma. We demonstrated that shikonin had prompt anti-tumor effect on osteosarcoma cells, which had no impact on cell cycle. This indicated that shikonin kills osteosarcoma cells directly rather than inhibiting proliferation. The majority of K7, K12, K7M3 and U2OS cell death induced by shikonin could be rescued by Nec-1, a specific inhibitor of necroptosis, while Z-VAD-FMK, a general inhibitor of apoptosis, had no obvious protective effect. As a potent necroptosis inhibitor, Nec-1 was previously believed not to inhibit apoptosis [7,14,21]. Thus the death of K7, K12, K7M3 and U2OS cells induced by shikonin could be considered as necroptosis. The degree of necrosis and apoptosis were further detected by flow cytometry with Annexin V and PI double staining. The results showed that necrotic cells were almost totally prohibited by pretreatment with Nec-1 before exposure of shikonin in K7 cells. Additionally, K7 cells at late apoptotic stage were also attenuated by Nec-1, indicating that part of those cells might be necrotic cells. This result is similar to previous data [21]. Therefore, shikonin could kill osteosarcoma cells quickly by inducing necroptosis at least in some osteosarcoma cell lines.

RIP1 and RIP3 are regarded as crucial modulators of necroptosis [8-10]. In our study, we found the protein levels of RIP1 and RIP3 were significantly increased after treatment with shikonin in a concentration dependent manner in some osteosarcoma cell lines including K7 and U2OS. These results indicated that shikonin induced cell death in some osteosarcoma cell lines including K7 and U2OS via RIP1 and RIP3 dependent necroptosis pathway. Moreover, other reports have also shown that necroptosis could be induced via modulating RIP1 and RIP3 [16,22-24]. Also, there were results different from ours. Moujalled, D. M. et al. demonstrated that TNF can activate RIP3 and cause necroptosis in the absence of RIP1 [25]. From the experiments in vivo, we also found that the protein levels of RIP1 and RIP3 in primary tumor tissues were increased in shikonin group compared with control group. It can be inferred that shikonin had anti-tumor effect in vivo probably by inducing necroptosis, just as the mechanism proved by the previous mentioned cell studies.

Interestingly, we found that in osteosarcoma 143B cells, which were previously detected less sensitive to shikonin compared with K7 and U2OS cells, cell death induced by shikonin could neither be reduced by Nec-1 nor by Z-VAD-FMK. We also found that RIP1 and RIP3 had no obvious change while caspase-3, caspase-6 and PARP were not activated after being treated with shikonin. Different from our results, Chang, et al. found that shikonin induces apoptosis through reactive oxygen species/extracellular signal-regulated kinase pathway and PARP was activated in 143B cells after being treated with shikonin for 24 hours [26]. It may be because the treatment time was different and need further study. As we know, the 143B cell line is a Ki-ras transformed TE85 [27,28] and not sensitive to shikonin. Maybe Ki-ras is a barrier to necroptosis. Interestingly, we also found the cell death of SaoS2 cells induced by shikonin could not be rescued by Nec-1(data not shown). U2OS is a p53-positive cell line while SaoS2 is a p53-null cell line [29]. We found the protein level of p53 was increased after treated with shikonin for 8 hours(data not shown). Maybe p53 is a regulator of necroptosis. Above-mentioned hypothesis is what our recent works focus on and needs further study.

The drug-resistance of cancer is associated with apoptotic pathway tightly, including overexpression of anti-apoptotic proteins, mutations of pro-apoptotic proteins and the loss of caspase [30-32]. In the clinic single-agent activity of methotrexate, cisplatin, doxorubicin and ifosfamide is approximately 40%, 30%, 40% and 30% respectively [33]. Combination chemotherapy yields slightly better results, but still approximately 40% of patients are not sensitive. The lung metastatic osteosarcoma also exhibits resistance to conventional chemotherapy. The 5-year survival rate for patients of osteosarcoma with metastasis is 20%, much lower than the corresponding survival rate for patients with localized disease (approximately 60%), and most death associated with osteosarcoma is the result of metastatic diseases. The specific mechanism of drug resistance of osteosarcoma might be associated with the activation of the Src and NF-κB pathway and the overexpression of anti-apoptosis genes [34-36]. Based on the results of this study, shikonin has strong anti-tumor effect on both primary and lung metastatic osteosarcoma by inducing necroptosis. As necroptosis undergo pathway independent of apoptosis, all the barriers set up in cancer cells to avoid apoptosis are no longer problems for necroptosis.

## Conclusions

Based on both in vivo and in vitro experiments, this study proved that shikonin had prompt but profound anti-tumor effect on both primary and metastatic osteosarcoma. The main mechanism of this effect might be inducing RIP1 and RIP3 dependent necroptosis. In order to develop its clinical use, in the future more studies are still required to evaluate the proper concentration and safety of shikonin, and shikonin related drug interactions in the treatment of osteosarcoma.

## Competing interests

The authors declare that they have no financial conflict of interests.

## Authors’ contributions

The work presented here was carried out in collaboration between all authors. ZC and YH defined the research theme, designed methods and experiments. ZF carried out the MTT assay, flow cytometry and Western blot, drafted the manuscript. LS, BD, YL, YH and FY carried out the in vivo experiments. YL, HZ and DZ analyzed the data. ZW and YL proofread the manuscript. All authors read and approved the final manuscript.

## Pre-publication history

The pre-publication history for this paper can be accessed here:

http://www.biomedcentral.com/1471-2407/13/580/prepub
